# Magnitude, associated factors, and antimicrobial susceptibility pattern of bacterial isolates among adult dental caries patients attending Hiwot Fana comprehensive specialized university hospital, Harar, Eastern Ethiopia

**DOI:** 10.1371/journal.pone.0278829

**Published:** 2023-02-15

**Authors:** Mandie Maru, Zelalem Teklemariam, Desalegn Admassu

**Affiliations:** School of medical laboratory science, College of health and medical sciences, Haramaya University, Harar, Ethiopia; University of Wolverhampton, UNITED KINGDOM

## Abstract

**Background:**

Dental caries is defined as a dynamic diet microbial disease of teeth, which results in localized dissolution and destruction of the mineralized tissues of the teeth. Dental caries develops when there is a susceptible tooth exposed to pathogenic bacteria in the presence of substrate. Under these conditions, the bacteria metabolize substrate to form acid, which decalcifies teeth. Dental caries is among the top oral health problem in both developing and developed nations affecting around 20–50% of the population globally.

**Objective:**

This study was conducted to assess the magnitude, associated factors, and antimicrobial susceptibility pattern of bacterial isolates among adult dental caries patients visiting Hiwot Fana specialized university hospital dental clinic from April 23 to-June 23, 2021.

**Methods:**

An institutional-based cross-sectional study was conducted among 290 study participants. Convenient sampling techniques were used to select the study participants. Data was entered into Epi-info version 7.2.4.0 and exported to Statistical Package for the Social Sciences version 20 for analysis. The result was explained by using summary measures of texts, tables, and graphs after analysis by using bivariate and multivariate logistic regression. Statistical significance was defined at a p-value of less than 0.05.

**Result:**

The overall magnitude of bacteria among dental caries patients was 68.3%. *S mutans* 74(37.4%) and *Lactobacillus* spp 58(29.3%) were the most predominant isolated bacteria. Lack of teeth brushing (AOR: 2.8, 95% CI:1.6, 4.6), the habit of chewing khat always (AOR:4.8, 95%CI:2.10,8.80), the habit of chewing khat sometimes (AOR: 3.8: 95% CI: 2.520,9.48) and consumption of soft drinks (AOR: 1.9, 95% CI:1.2,3.1) were significantly associated with bacterial dental caries. Almost, all bacterial isolates were susceptible to ceftriaxone and ciprofloxacin compared to Amoxicillin, Azithromycin, chloramphenicol, clindamycin, doxycycline, erythromycin, gentamycin, penicillin, tetracycline, vancomycin and tobramycin.

**Conclusion:**

Teeth brushing habit, consumption of soft drink and a habit of chewing khat affects dental health and they are associated with bacterial dental caries. Harari regional health bureau better to focus by giving health education to the community about dental caries based on identified associated factors with dental caries.

## 1. Introduction

Dental caries is defined as a dynamic diet microbial disease of teeth, which results in localized dissolution and destruction of the mineralized tissues. The disease can also be described as a microbial imbalance within the oral cavity in association with factors such as saliva, fluoride exposure, and diet [1]. In the formation of dental caries process, dental biofilm is the primary step. Dental biofilm is formed by bacteria sticking to each other and often adhering to a surface. The bacteria are embedded within a self-produced matrix of extracellular polymeric substance. In dental biofilm, *streptococcus mutans* is a major bacterium producing the extracellular polysaccharide matrix in dental biofilms [2].

Many variables can contribute to the caries process. Caries develops when there is a susceptible tooth exposed to pathogenic bacteria in the presence of substrate. Under these conditions, the bacteria metabolize substrate to form acid, which decalcifies teeth [3].

Dental caries is among the top oral health problem in both developing and developed nations affecting around 20–50% of the population globally and it is the main cause of tooth loss around the world [4]. Dental caries also significantly affects mastication and nutritional intake, speech, self-esteem, quality of life, and social interactions in the population [5].

The classic description of the cause of dental caries includes three factors: host, bacteria, and diet. Dental caries occurs when a susceptible tooth surface is colonized with cariogenic bacteria and a dietary source of sucrose or refined sugar is present. The frequent intake of sweet foods, dry mouth, and poor oral hygiene aggravate the chances of cavities in the teeth [6].

More than seven hundred different bacterial species have been detected in the oral cavity of humans [7]. For dental caries formation, the primary step is the formation of dental biofilm which means a layer of bacteria that can accumulate inside or on our teeth. The common predominant initial colonizers are oral *streptococci*, primarily from the *Streptococcus mitis* group followed by gram-positive rods like Lactobacillus spp, and especially Actinomyces species. However around 50% of dental caries-causing bacteria are there, which are not cultivable on artificial media like Prevotella, *Peptostreptococcus*, Bacteroidetes phylum, Fusobacterium, Capnocytophaga, Selenomonas, and Veillonella but it is possible to detect them using molecular techniques like 16S rRNA gene sequencing method and other staining procedures like Gram Stain, Endospore Stain, Capsule Stain (Negative staining) and Flagella Stain [8].

The most common method of caries detection is visual-tactile. Other non-invasive techniques for the detection of early caries have been developed and investigated such as Quantitative Light-induced Fluorescence, DIAGNOdent, Fibre-optic Transillumination, and Electrical Conductance [9].

The aims of this study are determining the prevalence, antimicrobial susceptibility patterns and factors associated with bacterial isolates from dental caries patients in the study area.

## 2. Methods and materials

### 2.1. Study area and period

The study was conducted in Harar town selected public hospital, Harar town, Harari Regional state from April 23 to June 23, 2021. Harar town is the capital city of the Harari region and is located 526 km east away from Addis Ababa, the capital city of Ethiopia. The total Harari region population (2019 projection based on the 2007 Census, CSA) was 257,000. Harar town has 19 kebeles, which comprise six districts. In the region, there are five hospitals (3 governmental and 2 private hospitals), 8 health centers, and 26 health posts. This study was conducted in Hiwot Fana comprehensive specialized University Hospital dental clinic from, Harar, Eastern Ethiopia, which is selected purposely, due to having high patients flow compared to other hospitals in the town.

Hiwot Fana comprehensive specialized University Hospital is currently the referral teaching hospital. [10, 11].

### 2.2. Study design

A hospital-based cross-sectional study was conducted among adult dental caries patients attending the dental clinic of Hiwot Fana comprehensive Specialized University Hospital.

### 2.3. Source population

- All adult patients attending at HFCSUH dental clinic.

### 2.4. Study population

All adult patients having dental caries attending at HFCSUH dental clinic during the study period.

### 2.5. Inclusion and exclusion criteria

#### 2.5.1. Inclusion criteria

- All ≥ 18 age group adult patients who visit the dental clinic of HFCSUH who are confirmed for dental caries by dentists

#### 2.5.2. Exclusion criteria

- Critically ill patients
- Patients who have taken antibiotics in the last 2 weeks or are currently taking was excluded
- Patients having mouth and the dental problem without caries as diagnosed by the dentist

### 2.6. Sample size determination

A single population proportion formula was used to calculate a sample size by taking the prevalence of bacterial dental caries from a study conducted in Debretabor comprehensive Hospital (78%) [12].


$$\text{n}=\frac{{\left({\text{Z}}_{\text{a}/2}\right)}^{2}\text{pq}}{{\text{d}}^{2}}$$

n = estimated sample size

Z = Z score for 95% confidence interval = 1.96

p = prevalence = 0.78

d = acceptable margin of error = 0.05

q = 1-p = 0.22

$$\text{n}=\frac{{\left(1.96\right)}^{2}\text{x}(0.78\text{x}0.22)}{{\left(0.05\right)}^{2}}=264$$

Including 10% contingency (non-response rate).

Then final sample size for this study = (10%x264) + 264 = 290

### 2.7. Sampling technique

A convenient sampling technique was used.

### 2.8. Data collection methods

#### 2.8.1. Face-to-face interview

Data was collected using face-to-face interviews using a pretest structured questionnaire by one trained clinical nurse, who is working at HFSUH dental clinic. The questionnaire was developed based on the research hypothesis and after reviewing different kinds of literature [13, 14]. The questionnaire contains two parts: Part I: socio-demographic variables like age, gender, marital status, educational level, income, and occupational group. Part 2: Associated factors like oral hygiene practice, and carbohydrate consumption. One BSc Laboratory technologist was supervising the activities of data collectors.

#### 2.8.2. Specimen collection

A sterile cotton swab took and dipped in a 1% glucose solution. The swab was then squeezed on the wall of the clean, dry, sterile test tube and pressed gently on the portion of the teeth cavity or scooping the cavity. This did by embedding the swab stick into the area of the carious lesion and lightly rotating 2–3 times, twisting the swab stick gently, then placing it into a 5 ml 1% glucose solution. Finally, labeled the tube with the date and specific codes. The swabs kept in a clean sterile tube and stored in an icebox till it reaches the side lab of microbiology at Haramaya university, college of health and medical science for processing [15].

#### 2.8.3. Bacterial culture and identification

Fifty μl of the sample inoculated into chocolate agar, blood agar, and de man, rogosa, and sharpe agar (MRS), plate then incubate at 37°C for 24–48 hours aerobically and 5% CO_2_ or using an anaerobic candle jar. Identification of bacteria did use colony characteristics, the gram reaction of the bacteria, and biochemical tests by following standard operational procedures (SOP). The gram-negative bacteria were identified by indole, H_2_S production in kligler iron agar (KIA) agar or TSI agar, motility, citrate, and urease. The gram-positive bacteria identified using their specific biochemical tests such as catalase, DNase, mannitol salt agar, bacitracin, optochin, indole, and urease. In addition, some discs used to identify pathogens like optochin and bacitracin. An amount of 50μl spread onto all media that was used by a sterile cotton swab and incubated in 5% CO_2_ for 48 hours at 37°C for isolation of *S*. *mutants* and 37°C for 24hr for isolation of lactic acid-producing bacteria. A colony count of more than 1×10^5^ cells/ml considered a positive sample [16].

#### 2.8.4. Antimicrobial susceptibility tests

The antibiotic susceptibility tests of the bacterial isolates did by using the Kirby-Bauer disc diffusion method. The sterilized nutrient broths prepared, and 3–5 colonies from the bacterial isolates inoculated by preparing suspension and by comparing its turbidity with 0.5 McFarland standards and plated onto a Mueller Hinton agar with and without sheep Blood by using a sterile cotton swab and squeeze the swab in the bottle and spreading it in all the directions over the surface of the agar to obtain a uniform growth. The antibiotics disc used, were ceftriaxone (30μg), ciprofloxacin (5μg), amoxicillin (25μg), erythromycin (15μg), penicillin (10μg), clindamycin (2μg), tetracycline (30μg), chloramphenicol (30μg), doxycycline (30μg), azithromycin (15μg), vancomycin (30μg), gentamycin (10μg), and tobramycin (10μg) placed over the agar and incubated at 37°C for 16–18 hrs. [17].

### 2.9. Study variables

#### 2.9.1. Dependent variables

- Bacteria isolates from dental caries patients

#### 2.9.2. Independent variables

- **Socio-demographic characteristics**: age, gender, marital status, income, educational level, occupation, residence.
- **Behavioral and health-related factors**: Teeth brushing habits, halitosis, consumption of sugars, frequent Consumption of soft drinks, hypertension, alcohol drinking, smoking cigarettes, chewing khat, and diabetes mellitus.

### 2.10. Data quality assurance

Data collector was trained for two days on data collection tools and how the sample collection procedures will be performed, by the principal investigator before the actual sample collection. The quality of data collection pretested at Jugal general hospital among 29 (10% of sample size) adult dental caries patients. Quality control measures were implemented throughout the whole process of the bacteriology laboratory work. Staining reagents, culture media, and antibiotic discs were checked for their normal shelf-life before use. All culture media was prepared according to the manufacturer’s instructions and sterility of the prepared culture media was checked by incubating 3–5% of the batch at 37°C overnight and observed for growth. Culture media, which showed any growth, were rejected and replaced by a new sterile batch, and all culture plates and antibiotic discs were stored at recommended refrigeration temperature (2–8°C) after preparation. Reference strains like *Streptococcus mutans* (ATCC 25175) *Lactobacilli uscasei* (ATCC 393), *Lactobacillus fermentum* (ATCC 9338), *S*. *aureus* (ATCC 25923), *E*.*coli* (ATCC 25922), *Pseudomonas aeruginosa* (ATCC 27853), and *K*. *pneumoniae* (ATCC 700603) were used [17].

### 2.11. Method of data analysis

Data was entered into EPI-Info version 7.2.4.0 and exported to Statistical Package for Social Science (SPSS) program version 20 (IBM Company). Descriptive statistics of different variables were performed using summary measures such as percentages and mean. Bivariate and multivariable logistic regression was carried out to identify the associated factors with bacterial dental caries. A variable with a p-value < of 0.25 on bivariate analysis was a candidate for the multivariable analysis. The multi-co-linearity test was carried out to observe the correlation between predictor variables using standard error and independent variables analysis. Finally, the adjusted odds ratio with 95% confidence intervals was used to determine statistical significance at a p-value < 0.05.

### 2.12. Ethical considerations

The study protocol was reviewed and approved by the College of Health and Medical Sciences Institutional Health Research Ethics Review Committee (IHRERC). Official letters of support were written to Hiwot Fana Comprehensive Specialized University Hospital (HFCSUH). Voluntary signed written consent was obtained from study participants to conduct the study after providing participants with information sheets. The study was explained to each participant, including the objectives, procedures, potential risks, and benefits of the study. The study participants were informed of their right to refuse or withdraw from the study at any time. Participants’ confidentiality of information was assured by excluding names and identifiers in the questionnaire. voluntary written and signed consent was obtained from all study participants.

## 3. Result

In this study, the magnitude of isolated bacteria among dental caries patients was 68.3%. In this study, a total of 290 dental caries patients were included with a response rate of 100%. The mean age of 33.44±12 (SD) years with a range of 18 to 80 years. The majority of study participants in this study were females (59%), Muslims (61%), urban residents (61%), and married (69%).

### 3.1 Socio-demographic characteristics of the participants

In this study, a total of 290 dental caries patients were included with a response rate of 100%. The mean age of 33.44±12 (SD) years with a range of 18 to 80 years. The majority of study participants in this study were females (59%), Muslims (61%) and urban residents (61%), married (69%), Government employees (26.6%), Secondary school and above (45.2%) and those having <1515 Ethiopian birr income per month were (63.8%) (Table 1).

**Table 1:** Socio-demographic characteristics of the dental caries patients attended at Hiwot comprehensive Fana specialized university hospital dental clinic (N = 290), Eastern Ethiopia from April to June 2021.

Variable	Categories	No (%)
Age (years)	18–27	108 (37.2)
	28–37	93 (32.1)
	38–47	51 (17.6)
	≥48	38 (13.1)
Gender	Male	119 (41)
	Female	171 (59)
Residence	Urban	177 (61)
	Rural	113 (39)
Marital status	Single	74 (26)
	Married	201 (69)
	Divorced	11 (4)
	Widowed	4 (1)
Level of education	Can’t read and write	64 (22.1)
	Read and write	32 (11)
	Primary school (1–8)	74 (25.5)
	Secondary school (9–12) and above	120 (45.2)
Types of occupation	Farmer	57 (19.7)
	Student	57 (19.7)
	Government employee	77 (26.6)
	Housewife	49 (16.9)
	NGO employee	20 (6.9)
	Private business	30 (10.3)
Level of income	<1515	185 (63.8)
	≥1515	105 (36.2)
Religions	Muslim	177 (61)
	Orthodox	65 (22.4)
	Protestant	24 (8.3)
	Catholic	24 (8.3)

### 3.2. Behavioral and health-related factors of the participant

The majority of the study participants had a habit of teeth brushing 186 (64%) and out of this, 153 (82%) of them had a habit of teeth brushing once per day. A total of 126 (43.4%) study participants consumed sugar, while 85 (29.3%) study participants consumed tea. 16 (5.5%) of the study participants were smokers, out of this, 9 (56.3%) of them were smokers once a day. 114 (39.3%) of the study participants chewed khat always, out of this, 26 (14.4%) of them chewed khat with sugar. A total of 6 (2.1%) and 10 (3.4%) of the study participants were diagnosed with diabetes and hypertension, respectively (Table 2).

**Table 2:** Behavioral and health-related factors of the dental caries patients attended at Hiwot Fana comprehensive specialized university hospital dental clinic (N = 290), Eastern Ethiopia from April to June 2021.

Variables	Categories	No (%)
A habit of teeth brushing	Yes	186 (64.1)
	No	104 (35.9)
Frequency of teeth brushing per day (n = 186)	Twice per day	19 (10.2)
	Once per day	153 (82.3)
	Once per week	14 (7.5)
Time for teeth brushing (n = 186)	Both in the morning and at night	19 (10.2)
	Brushing when I wake up in the morning	139 (74.7)
	Brushing after lunch	28 (15.1)
Material for teeth brushing (n = 186)	Modern tooth brush	119 (64)
	Mefakiya or traditional stick	67(36)
Consumption of sugar per week	Always	126 (43.4)
	Three days per week	15 (5.2)
	One day per week	74 (25.5)
	I don’t use sugar	75 (25.9)
Kind of sweet food used most	Candy	30 (10.3)
	Soft drink	58 (20)
	Chewing gum	17 (5.9)
	Tea	85 (29.3)
	Bread	80 (27.6)
	No	20 (6.9)
Smoking cigarette	Yes	16 (5.5)
	No	274 (94.5)
Frequency of smoking cigarettes (n = 16)	≤ Once per day	9 (56.3)
	≥ two times per day	7 (43.8)
Halitosis	Yes	27 (9.3)
	No	263 (90.7)
Consumption of Alcohol	No	279 (96.2)
	One day per week	11 (3.8)
A habit of chewing khat	Always	114 (39.3)
	Sometimes	66(22.8)
	Never at all	110(37.9)
Chewing khat with (n = 180)	With sugar	26 (14.4)
	With peanut	25 (13.8)
	With coffee	40 (22.3)
	With soft drink	39 (21.7)
	Alone	50 (27.8)
Consumption of soft drinks	Yes	137 (47.2)
	No	153 (52.8)
Frequency of consumption of soft drinks (n = 137)	Five days per week	43 (31.4)
	One day per week	94 (68.6)
Diabetic	Yes	6 (2.1)
	I don’t know	6 (2.1)
	No	278 (95.9)
Hypertension	Yes	10 (3.4)
	I don’t know	7 (2.4)
	No	273 (94.1)

### 3.3. Magnitude and type of bacterial isolates

**Fig 1:**
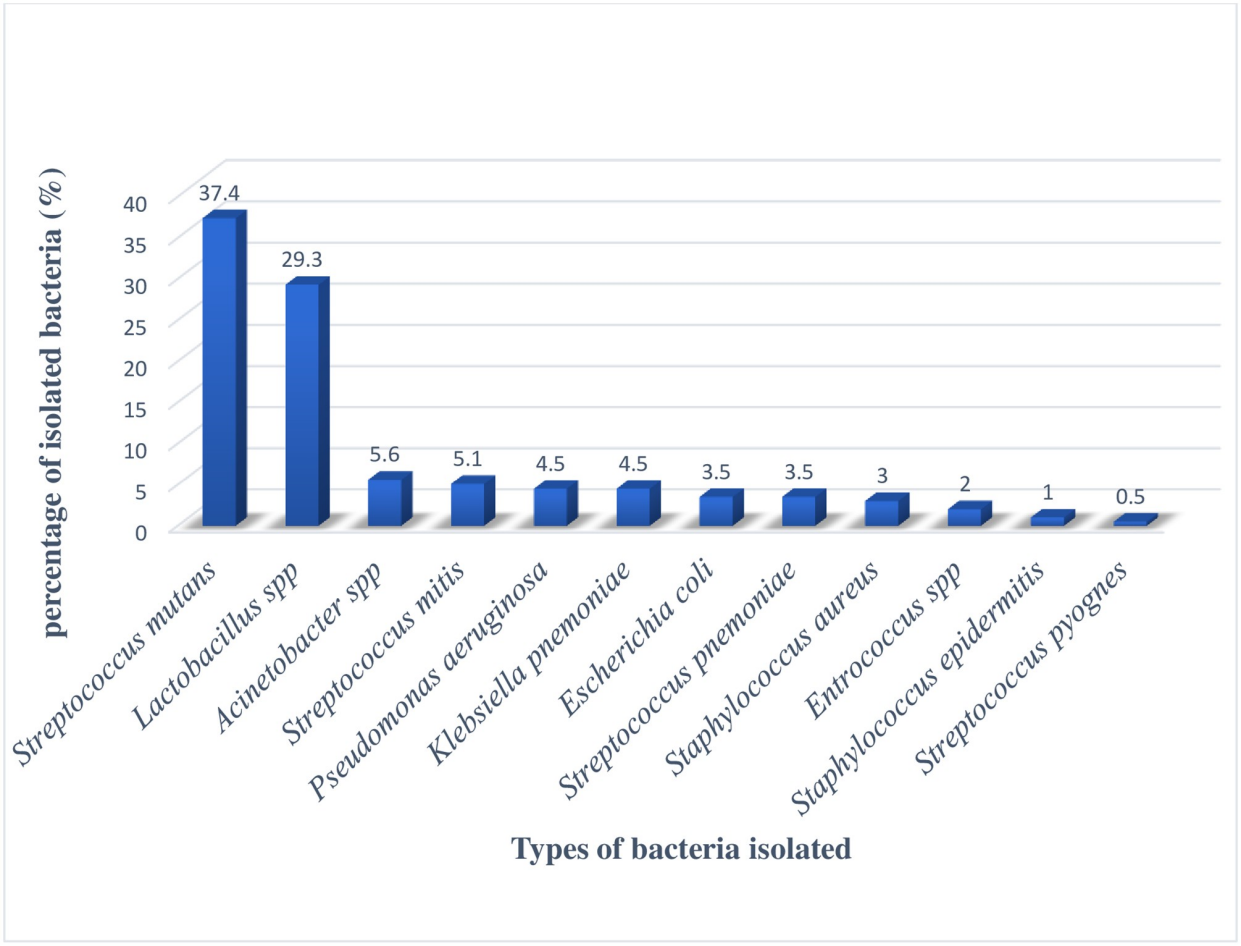
Distribution of isolated bacteria from dental caries patients attended at Hiwot Fana hospital.

In this study, the magnitude of isolated bacteria among dental caries patients was 68.3% (95% CI; 62.4,73.1). Gram-positive bacteria were the most common isolates 76.2% (95% CI; 69.4,83.1). *Streptococcus mutans* 37.4% (95% CI; 30.3,43.9) and *Lactobacillus* spp 29.3% (95% CI; 23.2,35.9) were commonly identified microorganisms (Fig 1).

### 3.3. Associated risk factors for bacterial dental caries

Tooth brushing habit, soft drink consumption, chewing khat habit, and sugar consumption, were candidates for multivariate analysis (p< 0.25) based on bivariate analysis.

Brushing teeth, chewing khat, and drinking soft drinks were all statistically associated with bacterial dental caries in a multivariate analysis. Those study participants who did not brush their teeth were more than 2.8 times more likely to develop bacterial dental caries than those who did brush their teeth (AOR: 2.8, 95% CI:1.6, 4.6). Those study participants who had a habit of chewing khat always were more than 4 times more likely to develop bacterial dental caries than non-khat chewers (AOR: 4.8, 95% CI:2.1, 8.8). Likewise, those study participants who had a habit of chewing khat sometimes were more than 3 times more likely to develop bacterial dental caries than non-khat users (AOR: 3.8, 95% CI:2.5, 9.5). Those study participants who had a habit of soft drinks were nearly 2 times more likely to develop bacterial dental caries than non-soft drink users (AOR: 1.9, 95% CI:1.2, 3.1) (Table 3).

**Table 3:** Factors associated with bacterial isolation among dental caries patients attended Hiwot Fana comprehensive specialized university hospital dental clinic (N = 290), Eastern Ethiopia from April to June 2021.

Variables	Categories	Bacteria isolated from culture		Crude OR (95%CI)	Adjusted OR (95% CI)	P-value
		Yes (%)	No (%)			
Level of income	<1515	122(65.9)	63(34.1)	1	1	
	≥1515	76(72.4)	29(27.6)	1.3(0.8,2.2)	1.6(1.0,2.8)	0.17
A habit of teeth brushing	Yes	48(25.8)	138(74.2)	1	1	
	No	60(57.7)	44(42.3)	2.1(1.2,3.5)	2.8(1.6, 4.5)	0.003*
A habit of chewing khat	Always	97(85.1)	17(14.9)	4.5(1.8,7.5)	4.8(2.1,8.8)	0.01*
	Sometimes	40(60.6)	26(39.4)	3.7 (2.4,8.6)	3.8(2.5,9.4)	0.02*
	Never at all	61(55.5)	49(44.5)	1	1	
Consumption of soft drinks	Yes	102(74.5)	35(25.5)	1.7(1.0,2.8)	1.9(1.1,3.0)	0.025*
	No	96(62.7)	57(37.3)	1	1	
Consumption of sugar per week	Always	83(65.9)	43(34.1)	1.1(0.5,1.9)	1.2(0.7,2.3)	0.16
	3 days per week	12(80)	3(20)	1.2(0.6,2.2)	1.4 (0.7,2.5)	0.14
	One day per week	55(74.3)	19(25.7)	1.1(0.4,2.7)	1.3 (0.7,2.8)	0.11
	I don’t use sugar	48(64)	27(36)	1	1	

Remark: -CI: Confidence interval,

*: statistically significant at p-value≤0.05; OR: Odd ratio

### 3.4. Antimicrobial susceptibility pattern of gram-positive isolated bacteria

In this study, most gram-positive bacteria were susceptible to ceftriaxone and ciprofloxacin. *Streptococcus pyognes* were susceptible to all antibiotics. *Acinetobacter* spp, *Enterococcus* spp, *Streptococcus pyognes*, *Staphylococcus aureus*, *Staphylococcus Epidermitis*, *and Streptococcus pnemoniae* were 100% susceptible to ciprofloxacin, except *streptococcus mitis*, all isolated gram-positive bacteria were 100% susceptible to ceftriaxone (Table 4).

**Table 4:** Antimicrobial susceptibility pattern of gram-positive isolated bacteria from dental caries patients attended at Hiwot Fana comprehensive specialized university hospital, dental clinic, Harar, Eastern Ethiopia from April to June 2021.

Bacterial isolates	Total No	Pattern	Antimicrobial susceptibility testing No (%)						
			CIP	AZT	T	C	CRO	VAN	E
*S mutans*	74	S	70(95)	52(70)	64(86)	42 (57)	74(100)	9 (12)	57(77)
		I	4(5)	16 (22)	-	21 (28)	-	39 (53)	13(18)
		R	-	6 (8)	10(14)	11(15)	-	26(35)	4 (5)
*Lactobacillus* spp	58	S	55(95)	37 (64)	48(82)	19(33.3)	58(100)	3(5)	45(77.3)
		I	-	11 (19)	5 (9)	16(27.7)	-	31(53)	11(18.3)
		R	3(5)	10 (17)	5 (9)	23(39)	-	24(42)	2(4.7)
*Enterococcus* spp	4	S	4(100)	2(50)	2(50)	1(25)	4(100)	1(25)	3(75)
		I	-	2(50)	2(50)	2(50)	-	3(75)	1(25)
		R	-	-	-	1(25)	-	-	-
*Streptococcus pyognes*	1	S	1(100)	1(100)	1(100)	1(100)	1(100)	-	1(100)
		I	-	-	-	-	-	-	-
		R	-	-	-	-	-	1(100)	-
*S aureus*	6	S	6(100)	5(83.3)	6(100)	5(83.3)	6(100)	-	6(100)
		I	-	1(16.7)	-	1(16.7)	-	2(33.3)	-
		R	-	-	-	-	-	4(66.7)	-
*S mitis*	10	S	8(80)	5(50)	8(80)	-	8(80)	2(20)	6(60)
		I	2(20)		-	-	2(20)	2(20)	4(40)
		R	-	5(50)	2(20)	10(100)	-	6(60)	-
*Staphylococcus epidermitis*	2	S	2(100)	-	2(100)	-	2(100)	-	2(100)
		I	-	2(100)	-	2(100)	-	2(100)	-
		R	-	-	-	-	-	-	-
*S pnemoniae*	7	S	7(100)	3(43)	7(100)	3(43)	7(100)	-	7(100)
		I	-	-	-	-	-	7(100)	-
		R	-	4(57)	-	4(57)	-	-	-
Bacterial isolates	Total No	Pattern	Antimicrobial susceptibility testing No (%)						
			PEN	DXL	AML	GEN	TOB	CD	
*S mutans*	74	S	-	-	46(62)	33 (45)	15(20)	31(42)	
		I	7 (9.5)	74(100)	22(30)	27(36)	37(50)	30(40)	
		R	67(90.5)	-	6 (8)	14 (19)	22(30)	13(18)	
*Lactobacillus* spp	58	S	3(4.8)	38 (65)	41(70)	39(68)	20(35)	25(43)	
		I	6(9.5)	12 (20)	9(16)	12(20)	32(56)	23(40)	
		R	49(85.7)	8 (15)	8(14)	7(12)	6(9)	10(17)	
*Enterococcus* spp	4	S	-	2(50)	3(75)	-	1(25)	3(75)	
		I	-	2(50)	1(25)	3(75)	-	1(25)	
		R	4(100)	-	-	1(25)	3(75)	-	
*Streptococcus pyognes*	1	S	1(100)	-	1(100)	-	1(100)	1(100)	
		I	-	1(100)	-	1(100)	-	-	
		R	-	-	-	-	-	-	
*S aureus*	6	S	-	5(83.3)	3(50)	2(33.3)	1(16.6)	3(50)	
		I	2(33.3)	1(16.7)	2(33.3)	4(66.7)	5(83.4)	3(50)	
		R	4(66.7)	-	1(16.7)	-	-	-	
*S mitis*	10	S	-	4(40)	6(60)	-	6(60)	6(60)	
		I	2(20)	6(60)	4(40)	6(60)	4(40)	4(40)	
		R	8(80)	-	-	4(40)	-	-	
*Staphylococcus epidermitis*	2	S	-	1(50)	1(50)	-	1(50)	1(50)	
		I	-	1(50)	1(50)	1(50)	1(50)	1(50)	
		R	2(100)	-	-	1(50)	-	-	
*S pnemoniae*	7	S	3(43)	3(43)		2(29)	6(86)	4(57)	
		I	-	4(57)	4(57)	5(71)	1(14)	3(43)	
		R	4(57)	-	3(43)	-	-	-	

AML: Amoxicillin; AZT: Azithromycin; CRO: Ceftriaxone; C: chloramphenicol; CIP: Ciprofloxacin; CD: Clindamycin; DXL: doxycycline; E: Erythromycin; GEN: Gentamycin; PEN: Penicillin; T: Tetracycline; TOB: Tobramycin; VAN: Vancomycin; S: Susceptable; I: Intermediate; R: Resistance

### 3.5. Antimicrobial susceptibility pattern of gram-negative isolated bacteria

The majority of gram-negative isolated bacteria were susceptible to ciprofloxacin and ceftriaxone but resistant to vancomycin and penicillin. *Acinetobacter* spp were completely susceptible to ciprofloxacin and ceftriaxone. *Escherichia coli* and *Klebsiella pnemoniae* were 100% susceptible to ceftriaxone (Table 5).

**Table 5:** Antimicrobial susceptibility pattern of gram-negative isolated bacteria from dental caries patients attended at Hiwot Fana comprehensive specialized university hospital, Harar, Eastern Ethiopia from April to June 2021.

Bacterial isolates	Total No	Pattern	Antimicrobial susceptibility N (%)						
			CIP	AZT	T	C	CRO	VAN	E
*Acinetobacter* spp	11	S	11(100)	5(45)	3(28)	3(28)	11(100)	-	4(36)
		I	-	6(55)	4(36)	4(36)	-	7(64)	7(64)
		R	-	-	4(36)	4(36)	-	4(36)	-
*E coli*	7	S	5(71)	3(43)	5(71)	-	7(100)	-	1(14.5)
		I	2(29)	4(57)		3(43)	-	3(43)	1(14.5)
		R	-	-	2(29)	4(57)	-	4(57)	5(71)
*P aeruginosa*	9	S	6(67)	-	-	-	6(67)	-	-
		I	1(11)	-	-	-	2(22)	1(11)	-
		R	2(22)	9(100)	9(100)	9(100)	1(11)	8(89)	9(100)
*K pnemoniae*	9	S	9(100)	4(44.4)	5(56)	2(22)	9(100)	-	3(33.3)
		I	-	3(33.3)	1(11)	5(56)	-	2(22)	3(33.3)
		R	-	2(22.3)	3(33)	2(22)	-	7(88)	3(33.3)
Bacterial isolates	Total No	Pattern	Antimicrobial susceptibility N (%)						
			PEN	DXL	AML	GEN	TOB	CD	
*Acinetobacter* spp	11	S	-	4(36)	2(18)	4(36)	5(45)	5(46)	
		I	5(45)	7(64)	5(45)	3(28)	2(18)	3(27)	
		R	6(55)	-	4(37)	4(36)	4(37)	3(27)	
*E coli*	7	S	-	5(71)	3(43)	4(57)	4(57)	2(29)	
		I	-	2(29)	1(14)	3(43)	-	4(57)	
		R	7(100)	-	3(43)	-	3(43)	1(14)	
*P aeruginosa*	9	S	-	1(11.1)	3(33.3)	4(44.4)	2(22)	4(44.4)	
		I	2(22)	1(11.1)	1(10.7)	2(32.3)	3(33.3)	-	
		R	7(88)	7(77.7)	5(56)	3(33.3)	4(44.7)	5(55.6)	
*K pnemoniae*	9	S		4(46)	7(78)	4(44)	6(67)	5(55.6)	
		I	2(22)	-	1(11)	5(56)	3(33)	2(22.2)	
		R	7(88)	5(54)	1(11)	-	-	2(22.2)	

AML: Amoxicillin; AZT: Azithromycin; CRO: Ceftriaxone; C: chloramphenicol; CIP: Ciprofloxacin; CD: Clindamycin; DXL: doxycycline; E: Erythromycin; GEN: Gentamycin; PEN: Penicillin; T: Tetracycline; TOB: Tobramycin; VAN: Vancomycin; S: Susceptible; I: Intermediate; R: Resistance

### 3.6. Multi-drug resistance pattern of bacterial isolated from dental caries patients

The overall magnitude of multi-drug resistance of the bacterial isolates from dental caries was 21/198 (10.6%). Almost all of the MDR bacterial isolates were resistant to vancomycin (100%). The high magnitude MDR were found on Pseudomonas *aeruginosa* (78%), *Streptococcus mitis* (40%), and *Escherichia coli* (43%) isolate. Multi-drug resistance was not found in the most commonly isolated bacteria (*Streptococcus mutans* and *Lactobacillus* spp) in this study.

Azithromycin, tetracycline, chloramphenicol, vancomycin, erythromycin, penicillin, doxycycline, and amoxicillin was found resistant in five (56%) of the *Pseudomonas aeruginosa* isolates. Azithromycin, chloramphenicol, vancomycin, and penicillin resistance were found in four (40%) of the *Streptococcus mitis* isolates. Chloramphenicol, vancomycin, and erythromycin resistance were found in two (29%) of the *Escherichia coli* isolates (Table 6).

**Table 6:** Multidrug resistance profile of bacterial isolates with a combination of resistant antimicrobials. From dental caries patients attended at Hiwot Fana Specialized comprehensive University Hospital, dental clinic, Harar, Eastern Ethiopia from April to June 2021.

Bacterial isolates	Number of isolates (%)		Combination of antimicrobials
*E coli* (N = 7)	3 (43%)	2 (29%)	C, VAN, and E
		1 (14%)	C, VAN, E and PEN
*P aeruginosa* (N = 9)	7 (78%)	5 (56%)	C, VAN, E, PEN, DXL, AML AZT and T
		2 (22%)	C, VAN, E, PEN, DXL, AML TOB, CD, AZT and T
*S aureus* (N = 6)	2 (34%)	1 (17%)	VAN and PEN
		1 (17%)	VAN, PEN and TOB
*S mitis* (N = 10)	4 (40%)		C, VAN, PEN and AZT
*S pnemoniae* (N = 7)	2 (29%)		C, VAN, and PEN
*K pnemoniae* (N = 9)	3 (33%)		VAN, PEN and DXL

AML: Amoxicillin; AZT: Azithromycin; C: chloramphenicol; CD: Clindamycin; DXL: doxycycline; E: Erythromycin; PEN: Penicillin; T: Tetracycline; TOB: Tobramycin; VAN: Vancomycin.

## 4. Discussions

Doing this research in this field had a great impact, for example it could be an essential information about which pathogen the most causative agent of dental caries and the like. In this study, the magnitude of bacteria identified in dental caries patients was 68.3% (95% CI; 62.4,73.1). This finding was consistent to the study conducted in Jimma (68.7%) [13], Brazil (68.5%) [18], China (67.5%) [16], and Srilanka (68.8%) [19]. However, it was lower than the study reported in Debretabor, Ethiopia (78%) [12], and Qatar (85%) [20]. These studies’ findings were higher than the study conducted in Gondar, Ethiopia (23.6%) and Nepal (62.5%) [15]. The difference could be due to the different amounts of consumption of sweet food, in knowledge and practice of oral hygiene level of khat intake, alcohol consumption and amount of smoking cigarette.

*Streptococcus mutans* was the first and most common bacteria isolated with 37.4% (95% CI; 30.3,43.9). This is not comparable to a study conducted in Jimma, Ethiopia (68.7%) [13]. And, it was also lower compared to a study conducted in Iraq (66%) (Hussein, 2020), India (90.75%), (88%), (42.5%) [21–23], respectively, and in Nepal (43.7%) [15], Nigeria (39.7%) [24] and higher in a study conducted in Uganda (23.5%) [25]. The disparity in prevalence could be due to the sensitivity and specificity of the detection method, it could be subjective during identification and sampling techniques that are employed.

*Lactobacillus* spp was the second most common isolated bacteria in this study, accounting for 29.3% (95% CI; 23.2,35.9), which was lower than studies conducted in Iraq at 50% [26] and India at 39% and 56.73% [22, 27] respectively, However, this study higher compared to a study conducted in India 9% [21] and Nigeria 6.1% [24]. The disparity in prevalence could be due to the sensitivity and specificity of the detection method, it could be subjective during identification and sampling techniques that are employed.

*Acinetobacter* spp 5.6% (95% CI; 2.5, 8.6) was detected at a low magnitude, but it was the first highly isolated gram-negative bacteria in this study. This finding is lower than in a Bangladeshi study (37.5%) [28]. *Pseudomonas aeruginosa* 4.5% (95% CI; 2.0,7.6) was second among gram-negative bacteria isolated in this study. This was lower than study reported from Iraq (8.3%) [29], India (12.5%) [28], and Nepal (32.1%) [15]. The disparity in prevalence could be due to the sensitivity and specificity of the detection method, it could be subjective during identification and sampling techniques that are employed.

The above bacteria that live in the mouth digest foods and other ruminants turning into acids due to the ability to ferment carbohydrates, survival, and growth under low pH conditions, and ability to synthesize extracellular and intracellular polysaccharides. The bacteria, acid, food debris and saliva combine to form plaque, which clings to the teeth. The acids in plaque dissolve the enamel surface of the teeth, creating holes in the teeth called cavities [30].

Bacterial dental caries has been associated with a variety of causes. The risk of developing bacterial dental caries increases by 2.8 in people who did not brush their teeth. This is supported by similar findings done in Jimma Ethiopia [13], and this also agrees with a study done at Debre Tabor [12]. Keeping oral hygiene have a great role in preventing bacterial dental caries, because the bacteria may not produce acids if food ruminant or debris is removed by teeth brushing [31]. The reason for this disparity could be the types of material for teeth brushing, time for teeth brushing, and way of teeth brushing.

Patients who drink soft drinks had a 1.9 times higher chance of developing bacterial dental caries than non-drinker. This agreed with a study done in Jimma, Ethiopia, [13]. This could be related to acid generation by cariogenic organisms that attach to teeth as a result of soft drink fermentation. The enamel of the tooth eventually deteriorated, resulting in tooth decay [15].

The odds of developing bacterial dental caries increase by 4.8 and 3.8 among those patients who chew khat always and occasionally respectively compared to those who hadn’t a habit of chewing khat. This not agreed with a study conducted in Jimma, Ethiopia showed, no association between chewing khat and dental caries and the p-value was 0.589 [32]. The difference might be due to the materials used during chewing khat (such as sugar, coffee, peanuts, and soft drinks), the amount of khat consumed per day, and the sample sizes used, and sampling methodologies used.

Increased antibiotic resistance is a global problem that accounts for 1.27 million deaths globally due to anti-microbial resistance. In poor nations, the reasons for antimicrobial resistance (AMR) are multidirectional, such as unlawful selling of antimicrobials, and empirical antibiotic treatment [33].

In this study, *streptococcus mutans* was susceptible to ceftriaxone 100%, ciprofloxacin 95%, tetracycline 86%, erythromycin 77%, chloramphenicol 57%, gentamycin 45% azithromycin 70% and amoxicillin 62%. This study was not in agreement with a study conducted in India [34] in which azithromycin was susceptible at 78%, and ceftriaxone was susceptible at 70% in Ethiopia [35]. This study also indicates *streptococcus mutants* was resistance for penicillin at 90.5%. This was not in agreement with a study conducted in India which reported penicillin resistance at 75%, gentamycin at 79%, chloramphenicol at 73%, and erythromycin at 75.9% [34]. The resistance disparity could be empirical antibiotic treatment and procedural subjectivity during sensitivity testing.

In this study *Lactobacillus* spp showed to be susceptible to ciprofloxacin 95%, gentamycin (68%), erythromycin 77.3% azithromycin 64%, but it was resistance to penicillin 85.7%. This study was similar report to a study done in Nepal [15], which found that ciprofloxacin was susceptible (94.27%), however, the current finding does not agree with a study conducted in Nepal [15], which found that gentamycin (51.85%) and erythromycin 49.49% were susceptible to *Lactobacillus* spp. The disparity in resistance and susceptibility might be due to unlawful selling of antimicrobials, empirical antibiotic treatment, and the use of antibiotics as prophylactic.

*Acinetobacter* spp found 100% susceptible to ciprofloxacin and ceftriaxone. This study was not comparable with a study conducted in Ghana found that it was susceptible to ciprofloxacin 70% and resistant to penicillin 70% [36].

*Pseudomonas aeruginosa* was 100% resistance to azithromycin, tetracycline, erythromycin, and chloramphenicol. But some isolates *Pseudomonas aeruginosa* were susceptible to ciprofloxacin (67%) and ceftriaxone (67%). This study is contradicted in a study conducted in India [34], which found that *Pseudomonas aeruginosa* was 70% susceptible to ceftriaxone and 85% susceptible to ciprofloxacin. A similar study conducted in Ghana [36], showed that *Pseudomonas aeruginosa* was 88% resistance to azithromycin, 92% resistance to ciprofloxacin, and 90% resistance to erythromycin. The above difference in antimicrobial susceptibility of bacterial isolates might be due to misused, easy availability of some drugs without prescription, and indiscriminate/prolonged use of common antibiotics.

Misuse of antimicrobial agents, inappropriate prescription practices, lack of patient adherence to antibiotic medication, inadequate patient education, limited diagnostic facilities, unauthorized antimicrobial sales, and a lack of appropriate drug regulatory mechanisms are just a few causes of antimicrobial resistance [37]. Antibiotic resistance is also caused by antibiotic destruction or modification, target alterations (target replacement, target site mutations, target site enzymatic alterations, target site protection, target overproduction, or target bypass), and reduced antibiotic accumulation due to decreased permeability and/or increased efflux. [38].

A total of 21 identified bacteria, or 10.6% of the total, were found to be resistance to various medicines in this investigation. In terms of drug combinations, it forms multi-drug resistance in two drugs 0.5%, three drugs 4%, four drugs 2.5%, eight drugs 2.5%, and ten drugs 1% of the tested panel. However, this study is contradicted by a study conducted in Nepal, which found that 267 isolates (82.15%) of 325 isolated organisms were MDR [15]. The combination of amoxicillin, doxycycline, and azithromycin drugs showed multi-drug resistance, but they are common prescribing drugs by dentists. So, this could impact the patient’s prognosis, waste money and time, and expose patients to other MDR bacteria. The difference in MDR variation could be inappropriate prescription practices in the study areas, empirical antibiotic treatment, use of antibiotics as a prophylactic, easy availability of some drugs without prescription, and indiscriminate/prolonged use of common antibiotics.

### 4.1 Limitation of the study

There were some limitations to this investigation. The sample size that was used in this study small so, the prevalence of bacteria may be underestimated and will not be the representative of the general population. Certain bacteria will not be identified at the species level, due to biochemical shortages. Furthermore, 50% of dental caries-causing bacteria cannot be cultured on artificial media without the use of molecular methods, therefore such bacteria may be ignored. Strictly anaerobic pathogenic bacteria such as Bacteroides, Prevotella, Fusobacterium, and Clostridium species were not detected due to a lack of laboratory facilities.

## 5. Conclusion and recommendation

Soft drinks, tooth brushing habits, and chewing khat were identified as associated factors with bacterial dental caries. *Streptococcus mutans* and *Lactobacillus* spp. were the most commonly isolated microorganisms. Most isolated bacteria were susceptible to ceftriaxone and ciprofloxacin. Harari Regional Health Bureau should be focus more on community health education about dental caries by focusing on identified factors, like tooth cleaning habits, limiting soft drink intake, and reducing the practice of chewing khat.

## Supporting information

S1 Dataset Patient information sheet, a questionnaire used for patient interviews, and the data that was collected. (XLSX)

## Abbreviations

AMR: Antimicrobial resistance
AOR: Adjusted odds ratio
ATCC: American Type Culture Collection
BSc: Bachelor of science
CLSI: Clinical Laboratory Standard Institute
COR: Crude odds ratio
CSA: Central Statistical Agency
HFCSUH: Hiwot Fana Comprehensive Specialized University Hospital
IBM: International Business Machines
IHRERC: Institutional Health Research Ethics Review Committee
MDR: Multi-Drug Resistance
MRS: de man, rogosa, and Sharpe agar
MSc: Master of science
NGO: Nongovernmental Organization
RNA: Ribosomal Nucleic Acid
SD: Standard Deviation
Spp: Species
SPSS: Statistical Package for Social Sciences
WHO: World Health Organization
